# *Staphylococcus aureus* β-hemolysin causes skin inflammation by acting as an agonist of epidermal growth factor receptor

**DOI:** 10.1128/spectrum.02227-23

**Published:** 2023-12-07

**Authors:** Yonggen Jia, Zhangchun Guan, Chenghua Liu, Minjun Huang, Jingjing Li, Jiannan Feng, Beifen Shen, Guang Yang

**Affiliations:** 1 Beijing Institute of Tropical Medicine, Beijing Friendship Hospital, Capital Medical University, Beijing, China; 2 Beijing Institute of Pharmacology and Toxicology, Beijing, China; 3 Cancer Institute, Xuzhou Medical University, Xuzhou, Jiangsu, China; Icahn School of Medicine at Mount Sinai, New York, New York, USA

**Keywords:** *Staphylococcus aureus*, β-hemolysin, EGFR, ADAM17, sphingomyelinase, skin inflammation

## Abstract

*Staphylococcus aureus* is a common cause of inflammatory skin diseases, with one of its virulence factors, β-hemolysin (Hlb), known to promote skin colonization. However, its direct involvement in skin inflammation is unclear. In this study, we identified that Hlb activated the epidermal growth factor receptor (EGFR) to induce skin inflammation, through its sphingomyelinase activity. We found that Hlb promoted the activity of A Disintegrin And Metalloproteinase 17 (ADAM17), which cleaves EGFR ligands and triggers EGFR phosphorylation. Furthermore, we discovered that Hlb increased the exposure of phosphatidylserine on the cell membrane, thus enhancing ADAM17’s sheddase activity. To address this, we developed a neutralizing monoclonal antibody against Hlb, which effectively attenuated *S. aureus*-induced skin inflammation by blocking EGFR activation. Our findings provide critical insights into the role of Hlb in inflammatory skin diseases, shedding light on the endogenous signal pathway that triggers this process.

**IMPORTANCE:**

*Staphylococcus aureus* is a Gram-positive opportunistic bacterium that is responsible for the majority of skin infections in humans. Our study provides important molecular insights into the pathogenesis of *S. aureus* skin infections and identifies a potential therapeutic target for the treatment of these infections. Our findings also indicate that β-hemolysin (Hlb) secreted by colonized *S. aureus* is a risk factor for epidermal growth factor receptor (EGFR)-related diseases by acting as an agonist of EGFR. The neutralized monoclonal antibody we have developed for the first time will provide a functional inhibitor of Hlb. This study provides important insights to better understand the relationship between the skin colonization of *S. aureus* and inflammatory skin diseases.

## INTRODUCTION

*Staphylococcus aureus* is a gram-positive, opportunistic bacterium that is responsible for the vast majority of bacterial skin infections in humans (1, 2). As one of the most abundant skin-colonizing bacteria, *S. aureus* causes several skin-inflammatory diseases, including impetigo, folliculitis, furuncles, abscesses, and wounds (3). *S. aureus* secretes several cytolytic or membrane-damaging hemolysins (α, β, δ, γ) (4 – 6). Among them, β-hemolysin (Hlb) is the only non-pore formation toxins that catalyze the hydrolysis of sphingomyelin to produce phosphocholine and ceramide (7). Sphingomyelin is abundant in the outer leaflet of the cell plasma membrane, where cholesterol forms lipid rafts, which serve as platforms for protein assemblies involved in membrane signaling and protein trafficking. Therefore, degradation of sphingomyelin mediated by Hlb is considered to be the major reason for erythrocyte lysis and proliferating human lymphocyte killing (8 – 10).

Hlb, further identified to belong to the DNase I superfamily, can act as a biofilm ligase to induce the formation of a nucleoprotein matrix in staphylococcal biofilms, which is important for *S. aureus* colonization (11). Moreover, Katayama et al. reported that Hlb promoted skin colonization of *S. aureus*, which is one of the characteristic features of several skin inflammatory diseases (12). However, whether Hlb can induce skin inflammation is still unknown.

Here, we identified that Hlb played an important role in the *S. aureus* skin infection. Hlb induced the activation of EGFR and subsequently caused skin inflammation. The inflammation induced by Hlb was dependent on the activities of sphingomyelinase but not the biofilm ligase of Hlb.

## RESULTS

### Hlb contributes to skin infection caused by *S. aureus*

**FIG 1:**
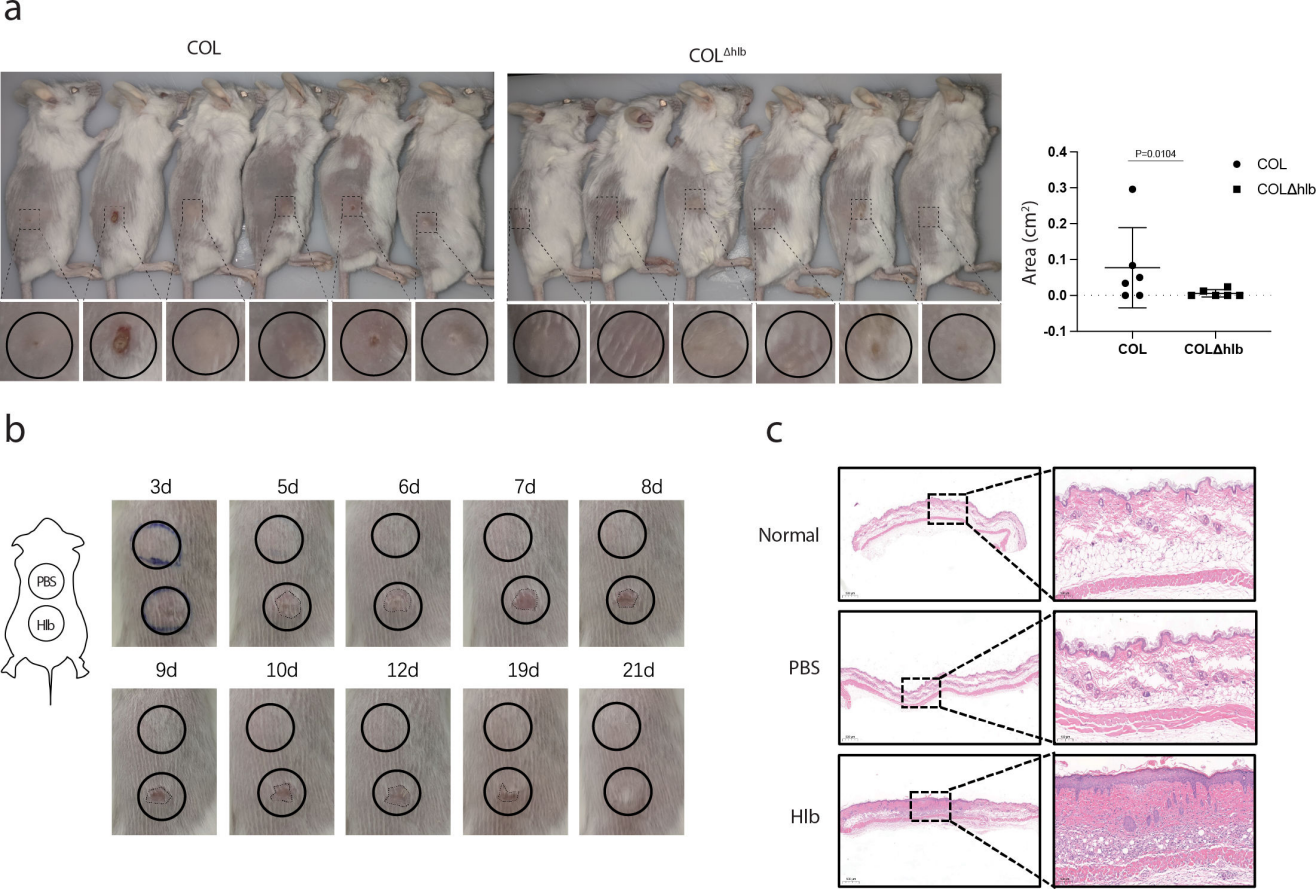
*S. aureus* beta-hemolysin (Hlb)-induced mouse skin inflammation. (a) Mice were infected with 50 µL COL or COL^Δhlb^ (3 × 10^7^ cells/mouse) (*n* = 6) by subcutaneous skin inoculation. Skin lesions were monitored daily. The image shows mice from both groups on the eighth day post-bacterial inoculation. The lesion sizes were measured and shown as mean ± standard deviation. (b) Monitoring lesions on the mice’s back skin on different days. Mice were administered with 50 µL recombinant Hlb proteins (0.4 mg/mL) or sterile PBS by subcutaneous injection (*n* = 3). The figure shows representative skin lesions within 3 weeks post-inoculation. (c) Pathological changes in mouse skin lesions were identified by H&E staining. Representative skin images were taken on the 8th day post-inoculation.

Hlb was reported to promote *S. aureus* colonization of the skin, yet its contribution to bacterial skin infection is still unknown. We first asked whether Hlb was involved in the skin infection caused by *S. aureus.* An hlb deletion strain (COL^Δhlb^) was generated from the *S. aureus* COL strain (13). COL^Δhlb^ strain and its parental *S. aureus* COL strain were subcutaneously injected into the flank of BALB/c mice, respectively. The ability of the two strains to cause abscesses in a mouse skin-infected model was evaluated. We found that the area of abscess caused by COL^Δhlb^ was smaller compared with the *S. aureus* COL strain at 8 days post-bacterial inoculation, and no dermonecrosis was observed in the COL^Δhlb^-infected group (Fig. 1a). To further investigate the role of Hlb in the *S. aureus* skin infection, the recombinant Hlb protein was subcutaneously injected into the lower back of mice. The area of the skin lesion was monitored at different time points post-infection. It was shown that Hlb protein caused an obvious skin lesion size compared with the PBS control, and the skin lesion lasted nearly 3 weeks (Fig. 1b). Skin lesion tissues were collected, and pathological alterations were determined by hematoxylin and eosin (H&E) staining. Compared with normal skin tissues, the skin tissues with Hlb injection showed obvious pathological changes, including the increased thickness of the epidermis and massive infiltration of inflammatory cells (Fig. 1c). Collectively, these data imply that Hlb can directly cause skin inflammation.

### Hlb-caused skin inflammation is dependent on the phosphorylation of EGFR

**FIG 2:**
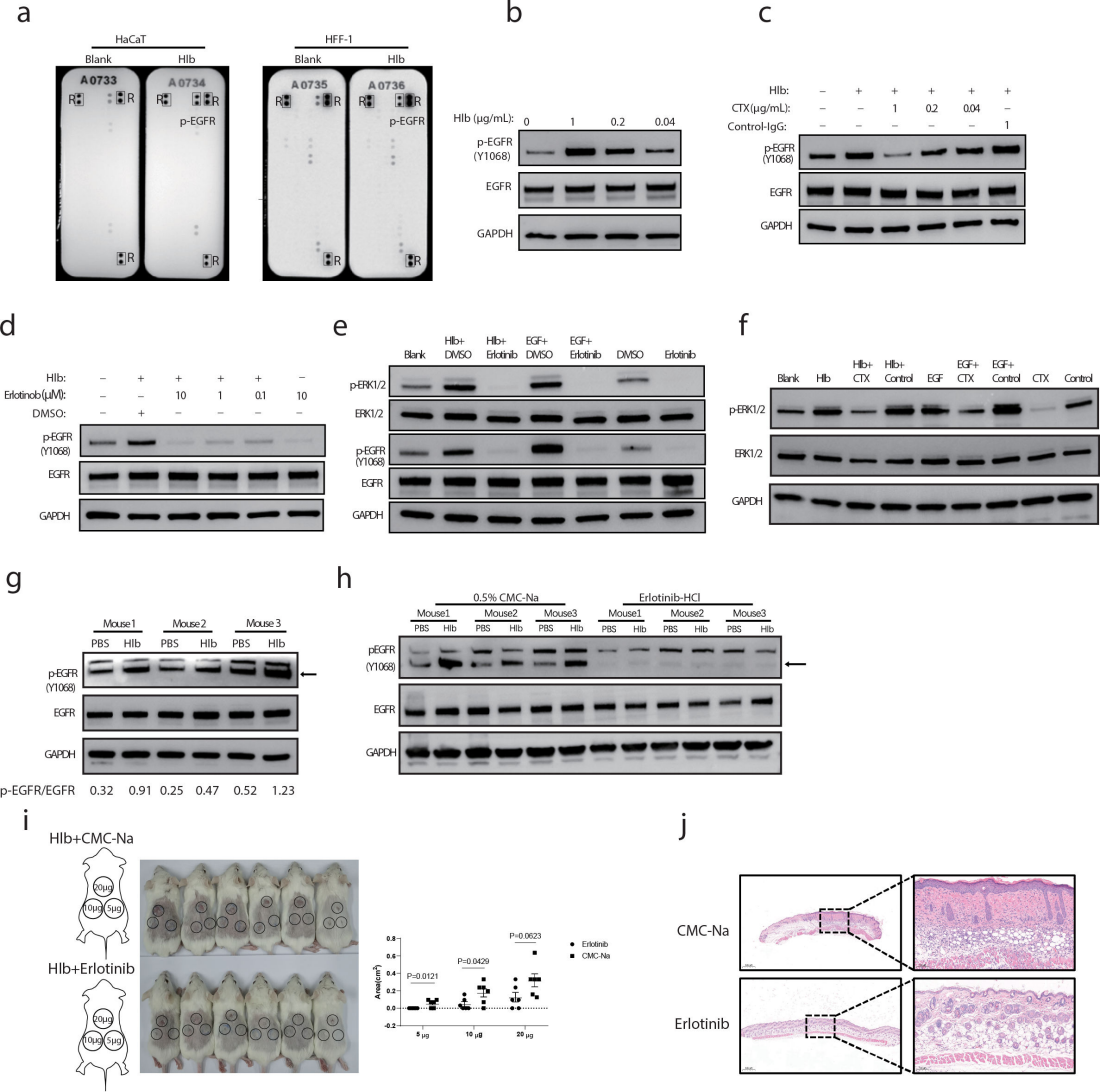
Hlb-induced skin inflammation in mice depends on EGFR activation. (a) Screening of RTK phosphorylation. HaCaT and HFF-1 cell lines were incubated with purified recombinant Hlb protein (1 µg/mL) for 10 min. The cells were lysed for the screening of phosphorylated RTKs with Human Phospho-RTK Array Kit (R&D Systems) that is a membrane-based sandwich immunoassay designed to detect the phosphorylation of 49 RTK simultaneously. The dots represent phosphorylated EGFR receptor, and the reference control was marked as p-EGFR and R, respectively. (**b**–d) Detection of Hlb-induced EGFR phosphorylation in HaCaT cells. HaCaT cells were treated with Hlb (1 µg/mL) for 10 min. Phosphorylation of EGFR was identified with anti-p-EGFR (Y1068) by immunoblotting, and GAPDH was used as a loading control (b). HaCaT cells were incubated with Hlb in the presence of EGFR-blocking antibody (Cetuximab) (c) or EGFR inhibitor—Erlotinib HCl (d) for 10 min. The cells were lysed, and EGFR phosphorylation was determined as above. (**e** and f) Detection of ERK phosphorylation induced by Hlb in HaCaT cells. HaCaT cells were incubated with Hlb in the presence of an EGFR-blocking antibody (Cetuximab, CTX) (e) or EGFR-inhibitor Erlotinib HCl (f) for 10 min. The cells were lysed, and ERK phosphorylation was identified with anti-p-ERK1/2 by immunoblotting. (**g** and h) Determination of EGFR phosphorylation induced by Hlb *in vivo* (*n* = 3). Mice were euthanized after subcutaneous back skin injection with Hlb for 10 min. The skin around the hydrogel site was cut off, and the total protein was extracted for the detection of EGFR phosphorylation (g). Erlotinib HCl or solvent was administered by gavage 1 h prior to Hlb injection. The skin total protein was extracted as above for the detection of phosphorylated EGFR (h). (i) Monitoring lesions on the mice’s back skin on the eighth day (*n* = 6). Erlotinib HCl or solvent was administered by gavage at a dose of 50 mg/kg body weight 1 h before Hlb subcutaneous injection. Thereafter, mice were administered erlotinib HCl daily, and the lesions were measured on the 8th day post-inoculation. Skin lesion sizes were measured using imageJ program. Data were presented as mean ± standard deviation and were analyzed by a two-tailed unpaired Student’s *t*-test. (j) Pathological changes in mouse skin lesions identified by H&E staining. The mice were euthanized to cut off the skin around the hydrogel site on the eighth day. The figure is a representative of the skins injected with 20 µg of purified recombinant Hlb protein at eight days post-inoculation.

Given that Hlb is a secreted toxin and has the potential to directly contact host cells, we further asked whether Hlb could activate the receptor tyrosine kinases (RTKs), the important signal molecules located at the cell membrane. Two human skin cell lines (HaCaT and HFF-1 cells) were incubated with Hlb for 10 min, respectively. In the concentration, we used for the following study, no cytotoxicity to the cultured HaCaT or HFF-1 cells was observed during Hlb incubation (Fig. S1). The activation of RTKs after Hlb treatment was determined by the Proteome Profiler Human Phospho-RTK Array Kit. Only one RTK, epidermal growth factor receptor (EGFR), was obviously significantly activated in the presence of Hlb (Fig. 2a).

The EGFR signaling pathway is important for maintaining skin homeostasis (14). Skin inflammation is the common adverse effect of EGFR inhibitors used in clinic (15). However, the upregulation of EGFR was involved in the development of several dermatologic diseases, including psoriasis (16). To validate whether Hlb could induce the phosphorylation of EGFR, we incubated HaCaT cells with different concentrations of Hlb proteins, and we found that Hlb induced EGFR phosphorylation in a dose-dependent manner (Fig. 2b). Two EGFR inhibitors [cetuximab (CTX) and Erlotinib Hydrochloride] were further used to verify the phosphorylation of EGFR induced by Hlb. We found both inhibitors successfully blocked Hlb-triggered EGFR activation (Fig. 2c and d). Furthermore, the phosphorylation of ERK1/2 at downstream of the EGFR signaling pathway was induced by Hlb, which was also blocked by both inhibitors (Fig. 2e and f). The Hlb-induced EGFR activation was also detected in the mouse skin tissues after Hlb injection (Fig. 2g and h), which was consistent with the data from those cultured cells.

We further investigated the role of EGFR activation in Hlb-induced skin inflammation. It was found that daily intragastric administration of the EGFR inhibitor Erlotinib Hydrochloride almost eliminated the skin inflammation caused by subcutaneous injection of a low dose of Hlb (5 µg/mouse) (Fig. 2i). Comparing with the solvent CMC-Na treatment group, no obvious pathological changes were found in the skin tissues after Hlb injection and daily Erlotinib administration (Fig. 2j). The area of skin lesion between Erlotinib Hydrochloride treatment group with a higher dose of Hlb injection (20 µg/mouse) was slightly smaller compared with the control group, though there was no significant difference between the two groups (Fig. 2i).

### Hlb-induced EGFR activation is dependent on Hlb’s sphingomyelinase activity

**FIG 3:**
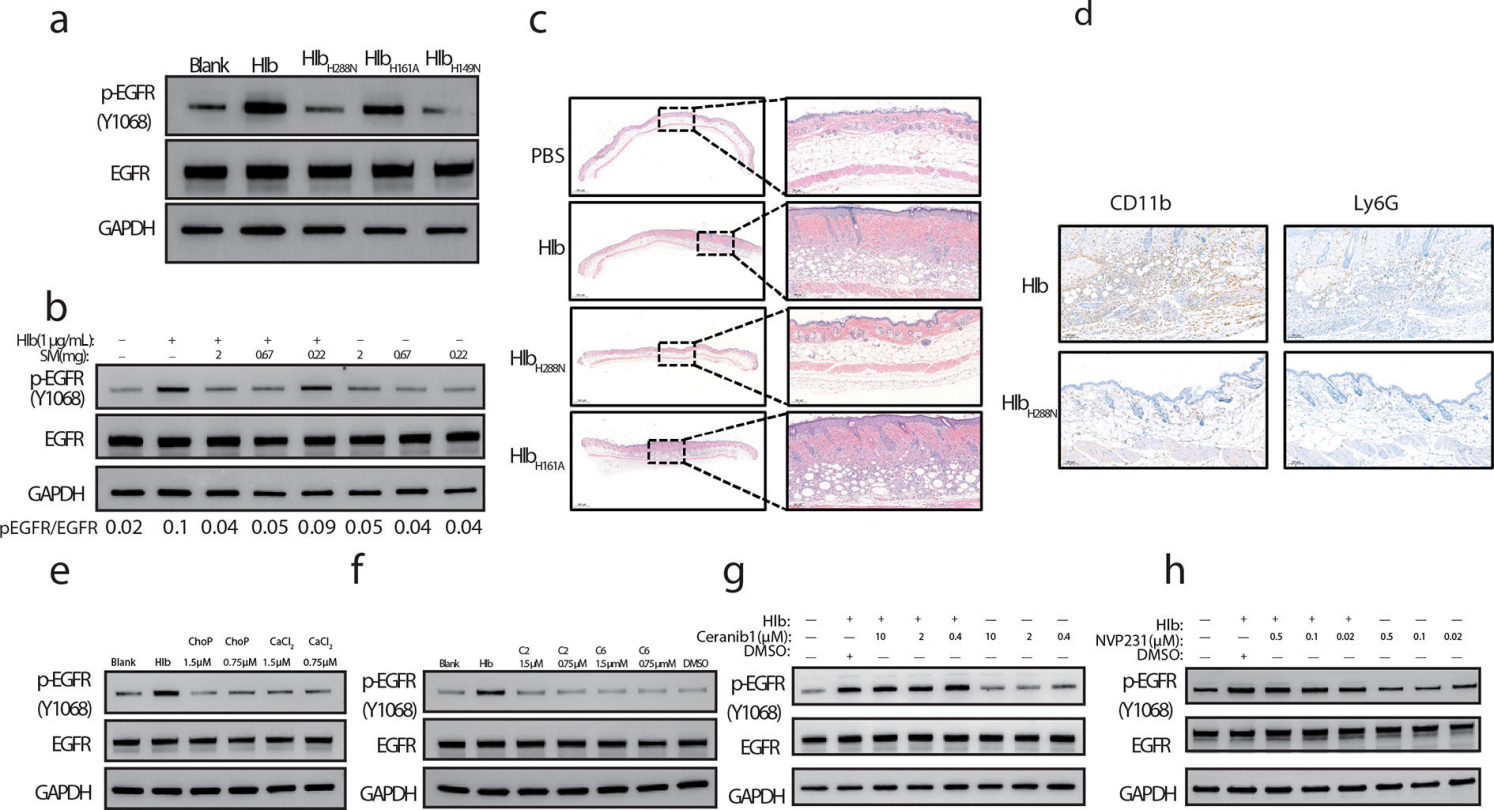
Sphingomyelinase activity is pivotal to Hlb-induced EGFR phosphorylation (a) HaCaT cells were incubated with Hlb, Hlb_H288N_ (1 µg/mL), Hlb_H161A_ (1 µg/mL), or Hlb_H149N_ (1 µg/mL) for 10 min. The cells were lysed, and EGFR phosphorylation was identified with anti-p-EGFR (Y1068) by immunoblotting. (b) HaCaT cells were incubated with Hlb in the presence/absence of various concentrations of sphingomyelin, and EGFR phosphorylation was determined as above. BALB/c mice were subcutaneously injected with Hlb (20 µg), Hlb_H288N_ (20 µg), or Hlb_H161A_ (20 µg). Pathological changes in mouse skin lesions (c) and massive infiltration of macrophage and neutrophil cells (d) were identified by H&E staining and immunohistochemical analysis at 8 days post-inoculation. Samples from Hlb-, Hlb_H288N_-injected skin were strained with CD11b and Ly6G. Representative images are shown. HaCaT cells were incubated with ChoP-CaCl_2_ (e) or C2 or C6 ceremide (f) for 10 min, and EGFR phosphorylation was determined as above. ChoP-CaCl_2_, calcium phosphorylcholine chloride. HaCaT cells were incubated with Hlb in the presence/absence of various concentrations of ceramidase inhibitor Ceranib1 (g) or ceramide kinase inhibitor NVP231 (h) and EGFR phosphorylation was determined as above.

It has been demonstrated that Hlb functions as a sphingomyelinase and a biofilm ligase. To interrogate which or both enzyme activities are involved in EGFR activation, sphingomyelinase-deficient mutant proteins (Hlb_H288N_ and Hlb_H149N_) and biofilm ligase-deficient mutant proteins (Hlb_H161A_) were expressed and purified, respectively (13). The phosphorylation of EGFR in HaCaT cells was subsequently determined after incubation with different Hlb mutants. Sphingomyelinase-deficient Hlb mutants rather than biofilm ligase-deficient mutants lost the capacity to simulate phosphorylation of EGFR (Fig. 3a). Accordingly, the level of Hlb-induced EGFR phosphorylation was decreased with the addition of sphingomyelin in a dose-dependent manner (Fig. 3b). As expected, the sphingomyelinase-deficient Hlb mutant totally lost the ability to induce mouse skin inflammation (Fig. S2a). Compared with sphingomyelinase-deficient Hlb mutants, the skin tissues injected with biofilm ligase mutant protein as well as Hlb wild-type protein showed obvious pathological changes, including the increased thickness of the epidermis and massive infiltration of macrophages and neutrophil cells (Fig. 3c and d; Fig. S2b and d).

Sphingomyelinase degrades sphingomyelin into ceramide and phosphocholine (17). Given that EGFR receptors are located at the cell membrane and sphingomyelin is one of the major components of the cell membrane, we asked if the degraded products of sphingomyelin mediated by Hlb were involved in the EGFR phosphorylation. The concentration of phosphocholine in the supernatant of A549 cell culture was increased after Hlb treatment, which was dependent on its sphingomyelinase activity (Fig. S2c). Some kinds of ceramide containing short-chain fat acids may be released from the cell membrane. However, the addition of phosphocholine or ceramides with short-chain fat acids (C2-ceramide and C6-ceramide), respectively, did not induce EGFR activation (Fig. 3e and f).

Ceramide is reported to be a signal molecule involved in the regulation of proliferation, differentiation, and apoptosis in epidermal keratinocytes (18, 19). Unlike phosphocholine, ceramide is mainly retained in the cell membrane after the degradation of sphingomyelin. Therefore, we wondered whether the signaling pathway mediated by the membrane ceramide was involved in the Hlb-induced EGFR phosphorylation. It was shown that neither the inhibitor of ceramidase nor the inhibitor of ceramide kinase could inhibit the phosphorylation of EGFR induced by Hlb (Fig. 3g and h). Taken together, the Hlb-induced EGFR phosphorylation depended on Hlb’s sphingomyelinase activity. The addition of its degradation products to the cell culture did not trigger EGFR activation.

### Hlb induces EGFR phosphorylation via ADAM17

**FIG 4:**
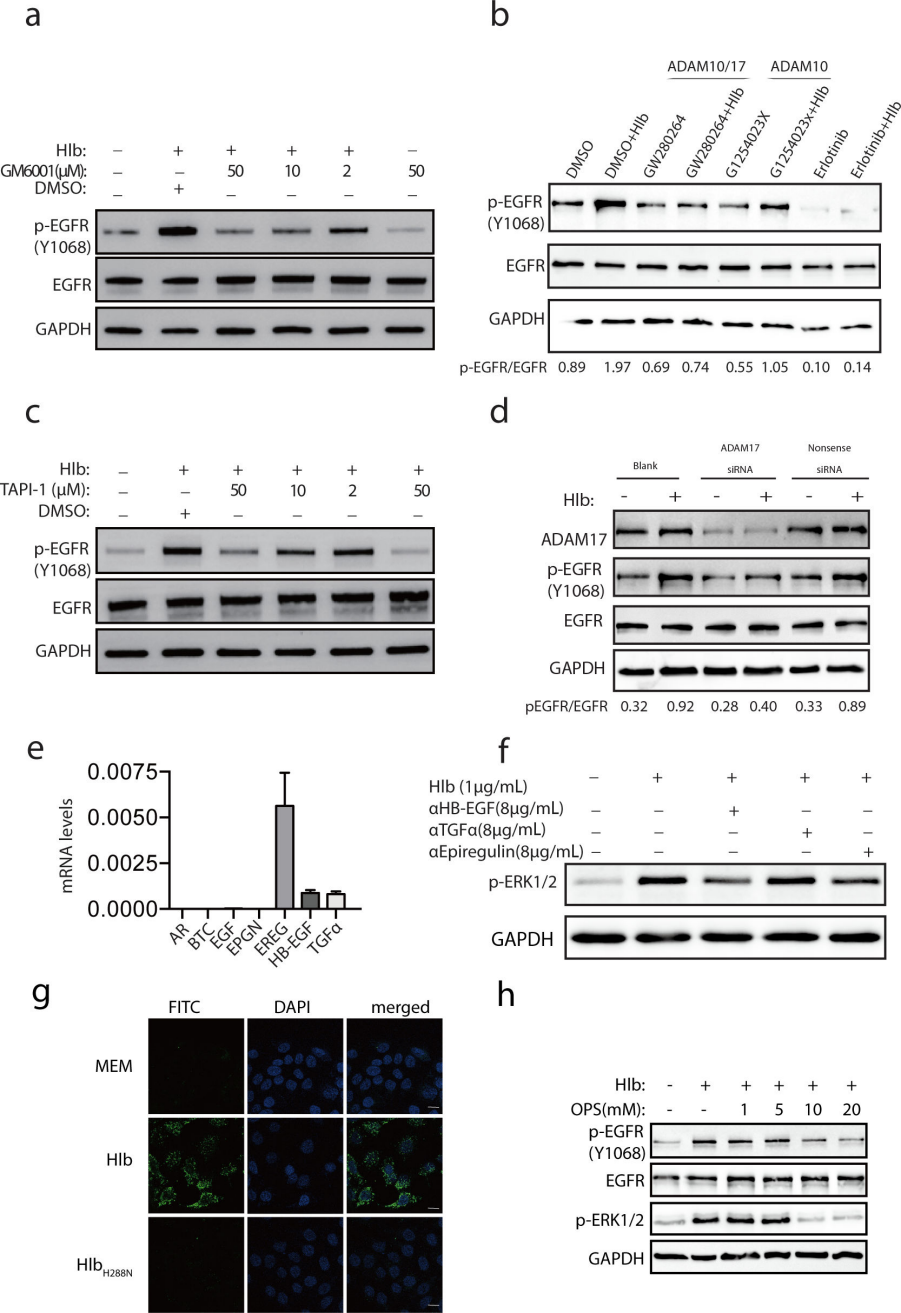
ADAM17 is involved in Hlb-induced EGFR activation. (a) A broad-spectrum metalloprotease inhibitor (GM6001) blocks Hlb-induced EGFR phosphorylation in a dose-dependent manner. (b) ADAM17/10 inhibitor (GW280264) but not ADAM10 inhibitor (GI254023X) significantly reduced Hlb-induced EGFR phosphorylation. (c) ADAM17-specific inhibitor (TAPI-1) significantly reduced Hlb-induced EGFR phosphorylation. Representative western blot images and corresponding data illustrating the expression of p-EGFR and ADAM17 in HaCaT cells with/without ADAM17 downregulation (d). The basal expression level of EGFR ligands (AR, amphiregulin; BTC, betacellulin; EGF, epidermal growth factor; EPGN, epigen; EREG, epiregulin; HB-EGF, heparin-binding; TGF-α, transforming growth factor-α) was assessed by RT-qPCR. Each value was normalized versus the corresponding mRNA level of GAPDH (e). HaCaT cells were incubated with Hlb protein (1 µg/mL) in the presence of blocking antibodies against epiregulin, TGF-α, and HB-EGF for 10 min. The cells were lysed, and ERK1/2 phosphorylation was detected with anti-p-ERK1/2 by immunoblotting. GAPDH was used as a loading control (f). HaCaT cells were incubated with MEM medium or in the presence of purified recombinant proteins Hlb (1 µg/mL) or Hlb_H288N_ (1 µg/mL) for 10 min and stained with Annexin V-FITC for detection of PS exposure. Scale bar, 10 µm (g). Hlb-induced EGFR phosphorylation was dose-dependently reduced by the addition of the competing phosphatidylserine head group (OPS). Representative western blotting of three independent experiments (h).

The activation of EGFR is triggered by the binding of its cognate ligands, including epidermal growth factor (EGF), transforming growth factor-alpha (TGF-α), heparin-binding EGF-like growth factor (HB-EGF), betacellulin (BTC), amphiregulin, epiregulin, and epigen (20). These ligands are expressed as type I transmembrane proteins; their extracellular regions (ectodomains) are usually shed by members of the ADAM (a disintegrin and metalloprotease) family (21). In addition, Hlb was reported to stimulate ectodomain shedding of syndecan-1 that is one of the substrates of ADAM17 (22). We assumed that Hlb activated EGFR by increasing the cleavage of the EGFR ligands. We then investigated if ADAMs were responsible for Hlb-induced EGFR activation. Cultured HaCaT cells were treated with Hlb with different concentrations of GM6001, a broad-spectrum metalloprotease inhibitor. We found that GM6001 significantly reduced Hlb-induced EGFR phosphorylation in a dose-dependent manner (Fig. 4a).

ADAM10 and ADAM17, ubiquitously expressed in mammalian cells, function as the major sheddases (23). ADAM10 inhibitor GI254023X and ADAM17 inhibitor GW280264 were incubated with HaCaT cells, respectively, in the presence of Hlb protein. We found that inhibitor GW280264 but not GI254023X inhibited the Hlb-induced EGFR phosphorylation (Fig. 4b). Besides, the ADAM17-specific inhibitor (TAPI-1) also blocked Hlb-induced EGFR phosphorylation (Fig. 4c). These data indicated that ADAM17, but not ADAM10, was involved in the Hlb-induced EGFR activation. This was further validated through the downregulation of ADAM17 expression with its specific siRNA. As expected, the Hlb-induced EGFR phosphorylation was significantly decreased in the HaCaT cells after downregulation of ADAM17 expression (Fig. 4d).

Four EGFR ligands (amphiregulin, epiregulin, TGF-α, and HB-EGF) have been reported as majorly cleaved by ADAM17 (24). To determine the expression levels of EGFR and its ligands in HaCaT cell lines, we performed RT-qPCR analysis. Among the seven ligands examined, we observed that epiregulin was predominantly expressed, followed by HB-EGF and TGF-α (Fig. 4e). Based on these results, we then tried to determine the level of these three ligands in the supernatants of cell culture after Hlb treatment by western blotting and ELISA. Attempts to detect any of those ligands were unsuccessful in our assay (data not shown), possibly due to their undetectable level in the cultured HaCaT cells. We next asked whether the blocking antibodies of epiregulin, HB-EGF, and TGF-α could inhibit Hlb-induced EGFR phosphorylation. The results showed that the blocking antibodies against epiregulin and HB-EGF effectively reduced the phosphorylation of ERK1/2 induced by Hlb. However, the blocking antibody against TGF-α did not show obvious inhibitory effects on ERK1/2 phosphorylation (Fig. 4f). These findings suggested that epiregulin and HB-EGF might play a crucial role in Hlb-induced EGFR activation. To examine the potential effect of Hlb on ADAM17 activity, we measured the levels of soluble TNFR1 (tumor necrosis receptor 1), a known substrate of ADAM17, in HaCaT cells treated with Hlb. Our findings revealed that incubation with Hlb led to an increase in the release of TNFR1 (Fig. S3). This observation suggests that treatment with Hlb may enhance the activity of ADAM17, leading to the cleavage of its substrates.

It was reported that the exposure of phosphatidylserine (25) on the cell surface was pivotal for ADAM17 sheddase activity (26). The binding of ADAM17 with PS exposed on the outer leaflet of the cell membrane caused ADAM17 conformational changes that facilitated the cleavage of substrates. Considering that Hlb is a sphingomyelinase that degrades the sphingomyelin that is the major component of the outer cell membrane, we detected whether the degradation of sphingomyelin led to the PS exposure. As expected, Hlb-induced PS exposure was detected by Annexin V after 10 min Hlb incubation (Fig. 4g). Concordantly, the addition of soluble PS in the cell culture efficiently blocked the Hlb-induced EGFR phosphorylation (Fig. 4h). In addition, flow cytometry analysis showed that inhibition of ADAM17 had no effect on the Hlb-induced PS exposure on the outer leaflet of the cell membrane (Fig. S4).

### Neutralized monoclonal antibody against Hlb we developed inhibits Hlb-induced EGFR activation and efficiently attenuated skin inflammation

**FIG 5:**
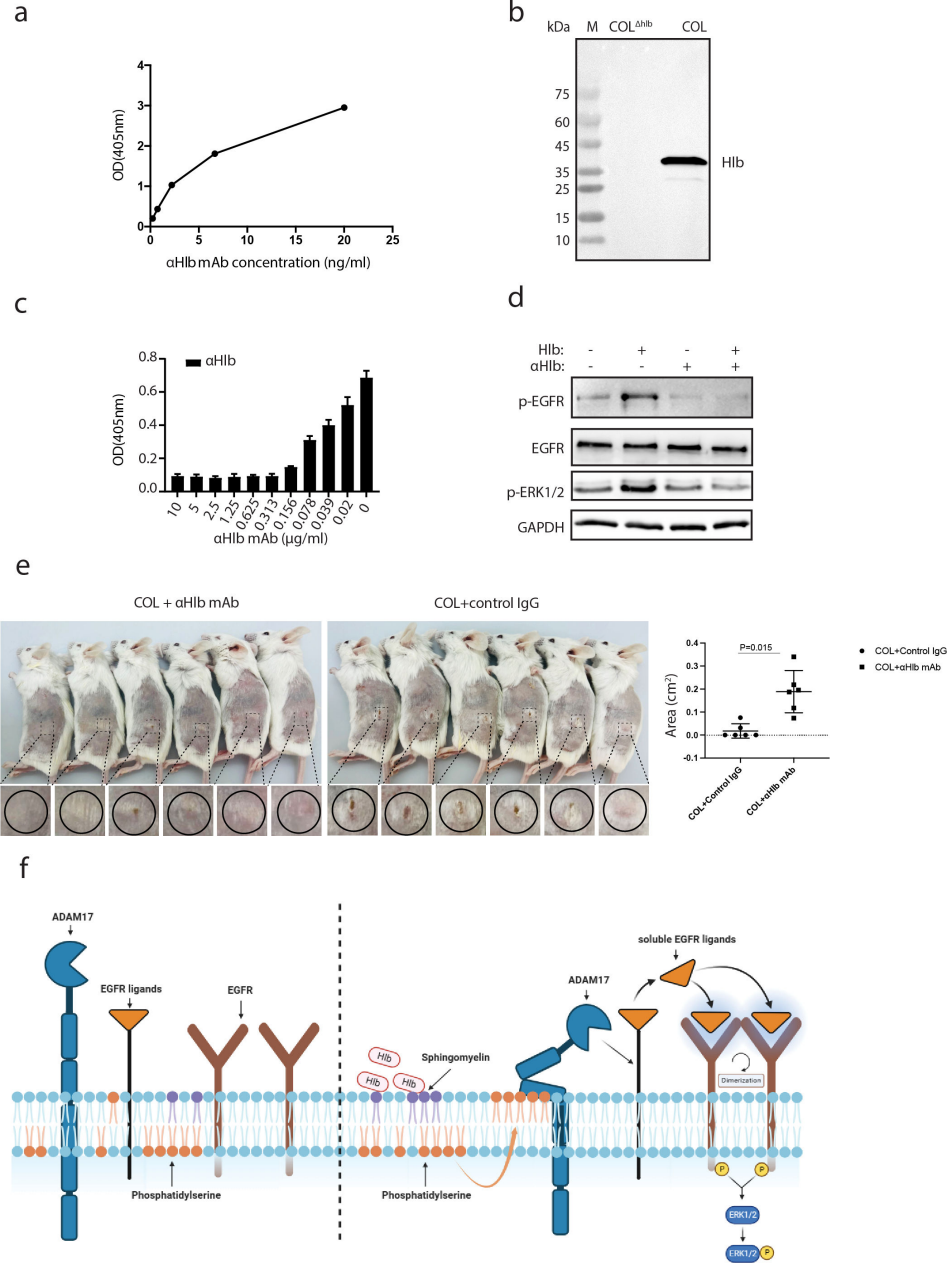
Hlb-specific monoclonal antibody inhibited Hlb-induced EGFR phosphorylation and attenuated *S. aureus* skin infection. (a) Determination of interaction of Hlb and its specific monoclonal antibody (anti-Hlb mAb, αHlb mAb) by ELISA. (b) Detection of the binding of αHlb mAb and Hlb secreted from *S. aureus* COL. (c) αHlb mAb blocked Hlb-induced red blood cell lysis in a dose-dependent manner. (d) αHlb mAb was pre-mixed with recombinant Hlb proteins to protein ratio 10:1. HaCaT cells were incubated with Hlb, αHlb mAb in the presence or absence of Hlb for 10 min. The cells were lysed and EGFR phosphorylation was identified with anti-p-EGFR (Y1068) by immunoblotting. (e) Mice were subcutaneously infected with *S. aureus* COL strain in the presence of αHlb mAb or unrelated mouse control IgG. Mice infected with COL strain with αHlb mAb developed significantly smaller lesions compared with mice infected with COL strain with control IgG antibody. The lesion sizes were measured and shown as mean ± standard deviation. (f) Proposed model for the activation of EGFR pathway induced by Hlb.

To our knowledge, no neutralizing monoclonal antibodies against Hlb have been reported. We, therefore, generated a novel-specific monoclonal IgG1 antibody against Hlb (anti-Hlb mAb) using mice hybridoma technology. The specific interaction between anti-Hlb mAb and Hlb protein was further determined by ELISA using purified recombinant Hlb protein (Fig. 5a). Moreover, anti-Hlb mAb specifically recognized the native Hlb protein secreted in the supernatant of *S. aureus* COL strain (Fig. 5b). Hlb is known for its ability to lyse red blood cells by degrading sphingomyelin enriched in sheep red blood cells. Therefore, we evaluated the ability of anti-Hlb mAb to block Hlb-induced lysis of erythrocytes. As expected, anti-Hlb mAb efficiently blocked the sheep red blood cells lysis mediated by Hlb (Fig. 5c). Furthermore, the Hlb-induced EGFR phosphorylation was inhibited with the addition of anti-Hlb mAb in cultured HaCaT cells (Fig. 5d). We further determined the effect of anti-Hlb mAb on the infection caused by different *S. aureus* strains. The skin infection caused by *S. aureus* COL and MW2 was significantly attenuated by the treatment of anti-Hlb mAb compared with the control group (Fig. 5e; Fig. S5). These results indicated that Hlb played a crucial role in the skin infection caused by *S. aureus*. In this process, Hlb acted as a sphingomyelinase to induce the exposure of PS from the inner to the outer cell membrane leaflet. This, in turn, activated ADAM17, resulting in an increased shedding of EGFR ligands. The released soluble ligands then bound to EGFR, initiating the activation of the EGFR signaling pathway. Activation of the EGFR pathway subsequently triggered various cellular responses downstream (Fig. 5f).

## DISCUSSION

In the present study, we identified that β-hemolysin obviously triggered the phosphorylation of EGFR and caused skin inflammation. EGFR has dual roles in maintaining skin homeostasis. Promotion or inhibition of EGFR phosphorylation induces skin inflammation. Cutaneous inflammatory rash is the most common adverse effect of EGFR inhibitors used for tumor therapy (27, 28). Nevertheless, it has been demonstrated that the upregulation of EGFR or its ligand expression plays an important role in the occurrence and development of chronic inflammatory skin disorders such as psoriasis (29). Epidemiological studies have shown that colonization of *S. aureus* is prevalent on the inflamed skin of patients with psoriasis (30, 31).

However, the contribution of *S. aureus* colonization to the development of psoriasis is still unknown. In our study, we identified that Hlb, an *S. aureus* virulence factor, stimulated EGFR phosphorylation and thus caused skin inflammation. Although bacteriophage ϕSa3 integrates into the *hlb* locus was up to 96% of human nasal *S. aureus* isolates, the expression of Hlb is common in strains with these strains by excision of phage ϕSa3 at a high frequency (32). It has been reported that Hlb is pivotal for *S. aureus* biofilm formation (12). Our results indicated that skin-colonizing *S. aureus* might be involved in the process of skin inflammatory disorders via secretion of Hlb.

ADAM17 is a crucial membrane-bound convertase responsible for the shedding of the ectodomains of various EGFR ligands, including amphiregulin, epiregulin, TGF-α, and HB-EGF (24, 32). In our study, we found that inhibition of ADAM17 activity using selective inhibitors or siRNA knockdown attenuated the Hlb-induced EGFR phosphorylation. We further demonstrated that three EGFR ligands (epiregulin, TGF-α, and HB-EGF) were predominantly expressed in HaCaT cells. However, attempts to detect these ligands in the cell culture using ELISA or western blot yielded undetectable levels. To assess the impact of Hlb treatment on ADAM17 activity, we measured the levels of TNFR1, a well-known ADAM17 substrate, in the cell culture (22). We observed that the level of soluble TNFR1 increased after Hlb treatment. These findings suggested that Hlb might enhance the activity of ADAM17, leading to increased EGFR phosphorylation.

In quiescent cells, mostly phosphatidylcholine and sphingomyelin are organized at the outer leaflet of the plasma membrane, while the PS and phosphatidylethanolamine are in the inner leaflet. The exposure of PS on the outer leaflet cell membranes increased the shedding of EGFR ligands mediated by ADAM17 (26). Our data demonstrated that Hlb induced the exposure of PS on the outer leaflet cell membranes. Moreover, we found that the phosphorylation of EGFR triggered by Hlb was correlated with exposure to PS. The addition of exogenous PS blocked the Hlb-induced EGFR phosphorylation. All these results suggested that the exposure of PS on the outer cell membrane triggered by Hlb increased the shedding of EGFR ligands of ADAM17 and subsequently induced EGFR phosphorylation. How Hlb induces PS exposure needs to be investigated in the future.

Hlb could function as both a sphingomyelinase and a biofilm ligase. Our results revealed that the sphingomyelinase-deficient mutant protein could not trigger the exposure of PS and the phosphorylation of EGFR. Moreover, sphingomyelin efficiently attenuated the activation of EGFR phosphorylation induced by Hlb. These results suggested that the Hlb-induced EGFR phosphorylation was dependent on the activities of sphingomyelinase rather than biofilm ligase.

In our experiment, the Hlb concentration we used did not induce any detectable signs of cytotoxicity in keratinocytes but significantly increased the phosphorylation of EGFR. Moreover, the administration of erlotinib hydrochloride efficiently attenuated skin inflammation caused by a low dose of Hlb (5 µg/mouse), which suggested that the activation of the EGFR pathway might play a major role in the skin inflammation caused by a low dose of Hlb. Although the concentration of Hlb in *S. aureus* biofilm *in vitro* was estimated to be above 15,000 µg/mL (33), the physiological concentration of hlb *in vivo* remained challengeable to be determined. Considering the quantity of *S. aureus* in infection and the rapid distribution of Hlb protein, we reasoned that the physiological concentration of Hlb *in vivo* should be significantly lower than the estimated concentration *in vitro*. Nevertheless, the effect of cytotoxicity of Hlb might not be ruled out in this process.

It has been previously reported that erlotinib, an oral tyrosine kinase inhibitor of EGFR, increases the expression of chemokines in primary human keratinocytes. This, in turn, leads to the infiltration of inflammatory cells and lymphocytes. Furthermore, erlotinib has been shown to decrease the production of antimicrobial peptides and skin barrier proteins, potentially resulting in bacterial colonization, particularly by *S. aureus* (34). In addition, a case report by Grenader et al. (25) described a patient with non-small-cell lung cancer who developed *S. aureus* bacteremia secondary to severe erlotinib skin toxicity. These reports suggested that erlotinib, as an EGFR inhibitor, might impair the innate host defense mechanisms, thereby increasing the risk of bacterial infection. However, erlotinib-induced skin inflammation was not observed in our murine model. Furthermore, treatment with erlotinib decreased the infection caused by recombinant Hlb protein. These findings suggested that the effects of erlotinib on skin inflammation and bacterial infection might vary depending on the experimental model and specific factors involved.

Bacterial cell wall-associated and secreted proteins and cell wall components of *S. aureus* detected by the innate immune system have been shown to be inflammatory (35). In our study, we found that the specific antibody targeting Hlb was unable to completely block the skin infection caused by *S. aureus*. In addition, even in the absence of Hlb, the Hlb deletion strain was still capable of causing skin infection. These results suggested that, in addition to Hlb, other virulent factors also contributed to the skin inflammation caused by *S. aureus*. But despite this, the decreased skin inflammation caused by the *hlb* deletion strain compared with its parental strain in our study implied that Hlb played an important role in this process.

Until now, there has been no reported neutralized monoclonal antibody against Hlb or specific inhibitors of Hlb. We developed a novel neutralized monoclonal antibody in this study, which could significantly inhibit the Hlb-induced EGFR phosphorylation. Upregulation of EGFR pathway signaling is a common event in several kinds of chronic diseases, especially in cancers such as non-small-cell lung cancer (NSCLC), metastatic colorectal cancer, and glioblastoma (36). Several EGFR-targeted therapeutics, such as monoclonal antibodies and tyrosine kinase inhibitors, have been approved for clinical use. As Hlb stimulated the phosphorylation of EGFR, the production of Hlb by colonized *S. aureus* might be a risk factor though the function of Hlb in those chronic diseases had not been investigated.

Taken together, our findings revealed that Hlb secreted by *S. aureus* triggered the EGFR signaling pathway, which not only provides the crucial mechanism of how Hlb induces skin inflammation but also offers a potential strategy for the management of *S. aureus* infection and EGFR-related diseases.

## MATERIALS AND METHODS

### Reagents and antibodies

The human Phospho-RTK Array Kit was obtained from R&D Systems. Phosphorylserine (OPS), sphingomyelin, and ADAM10 inhibitor (GI254023X) were from Sigma-Aldrich. Erlotinib Hydrochloride, a broad spectrum matrix metalloprotease (MMP) inhibitor GM6001, and ADAM17 inhibitor TAPI-1 were from Selleck. ADAM17/10 inhibitor (GW280264X) was from TargetMoI. EGFR-blocking antibody Cetuximab, ceramidase inhibitor Ceranib1, and ceramide kinase inhibitor NVP231 were from MCE. Annexin V-FITC from BD Bioscience. Anti-GAPDH, anti-EGFR, anti-phosphoEGFR, anti-ERK1/2, anti-phosphoERK1/2, and anti-TACE (ADAM17) were from Cell Signaling Technology (1:1,000). Goat anti-rabbit IgG-HRP and goat anti-mouse IgG-HRP were from Thermo Fisher Scientific (1:4,000). Blocking and neutralizing antibodies against human epiregulin, HB-EGF, and TGF-α antibodies were from R&D Systems (0.8 µg/mL). Additional antibodies used for flow cytometry were as follows: Brilliant Violet 605 anti-mouse CD11c (N418), FITC anti-mouse CD45 (30-F1), PerCP/Cyanine5.5 anti-mouse Ly-6G/Ly-6C (Gr-1) (RB6-8C5), PE anti-mouse F4/80 (BM8), and APC-anti-mouse/human. CD11b (M1/70) was from Biolegend (1:100).

### Cell culture

HaCaT cells (obtained from China Infrastructure of Cell Line Resources, 1101HUM-PUMC000373) were cultured in minimum essential medium (MEM) supplemented with 10% fetal bovine serum (FBS), 100 U/mL penicillin and streptomycin at 37°C, 5% CO_2_ in air atmosphere.

Human foreskin fibroblast (HFF-1 cells from ATCC SCRC-1041) cells were cultured in Dulbecco’s modified Eagle’s Medium (DMEM) supplemented with 10% FBS, 100 U/mL penicillin and streptomycin at 37°C, 5% CO_2_ in air atmosphere.

### Protein production

Recombinant wild-type Hlb, sphingomyelinase mutant, and biofilm ligase mutants were purified as previously described (13). In brief, the *hlb* gene (Gene ID: 3238648) was PCR amplified from the genome of *S. aureus* COL and cloned into the expression vector pET-28(a). The site-specific mutant genes were obtained by overlapping PCR. Recombinant proteins were expressed in *E. coli* (BL21) and purified by using Ni Sepharose affinity chromatography. Purified proteins were passed through the Pierce High-Capacity Endotoxin Removal Resin (Thermo Fisher Scientific) to remove endotoxins.

### RT qPCR

Total RNA was isolated from HaCaT cell lines using Trizol. After DNase treatment and retro-transcription, real-time qPCR was performed according to the standard protocols with commercially available kits (CWBIO Corp, Beijing, China). The primer pairs specific for various EGFR ligand genes used in our experiments were described in previous studies (37). A triplicate sample was analyzed in each group. GAPDH was used to normalize gene expression.

### The murine model of skin inflammation

BALB/c mice (female, 6–8 weeks old), C57BL/6N mice (female, 6–8 weeks old), and BALB/c Nude mice (female, 6–8 weeks old) were purchased from Vital River Company (Beijing, China) and housed under specific pathogen-free (SPF) conditions in filter-top cages, and sterile water and food were provided. For the bacterial skin infection, mice were injected subcutaneously in their flank with 50 µL of 3 × 10^7^ cfu of the *S. aureus* COL or COL^Δhlb^ cell suspension. For the Hlb protein inoculation, mice were administered with 50 µL of various concentrations of purified recombinant Hlb protein (0.4 mg/mL, 0.2 mg/mL, or 0.1 mg/mL) by subcutaneous injection in their lower back skin. For the antibodies mixed with bacterial skin infection, mice were injected subcutaneously in their flank with 50 µL of 6 × 10^7^ cfu of the *S. aureus* COL and MW2, respectively. The overall health of the mice was monitored on a daily basis by checking for any lesions and sacrifices at the endpoint of each experiment.

### Screening of human receptor tyrosine kinase phosphorylation

HaCaT or HFF-1 cells were incubated with purified recombinant Hlb proteins (1 µg/mL) for 10 min. The cells were washed once with cold PBS and lysed for the screening of phosphorylated RTKs with Human Phospho-RTK Array Kit (R&D Systems) according to the manufacturer’s instructions.

### Western blot analysis

HaCaT or HFF-1 cells were plated in 12-well plates (3 × 10^5^ cells/well) and treated with 1 µg/mL of purified recombinant Hlb protein or its mutants for 10 min on the second day. In some experiments, prior to Hlb protein (1 µg/mL) treatment, the cells were pre-treated with one of the following inhibitors: Erlotinib HCl, Cetuximab, GM6001, GI254023X, GW280264, TAPI-1, Ceranib1, or NVP231 for 30 min. Following Hlb treatment, the cells were immediately placed on ice and washed once with ice-cold PBS, and homogenized in 100 µL cold RIPA lysis buffer containing protease and phosphatase inhibitors. For the detection of the EGFR phosphorylation in mice skin tissue, the mice were euthanized after their back skin was subcutaneously injected with 50 µL Hlb (0.4 mg/mL) for 10 min, the skin around the hydrogel site was cut off and ground to a fine powder under liquid nitrogen. Resuspend the skin tissue with cold RIPA lysis buffer containing protease and phosphatase inhibitors. The samples were resolved on 12% SDS-PAGE and electroblotted onto PVDF membranes. Anti-EGFR, anti-Phospho-EGFR (Y1068), anti-ERK1/2, anti-Phospho-ERK1/2 and anti-GAPDH, anti-ADAM17 (Cell Signaling Technology) were used. Finally, the blots were analyzed with an enhanced chemiluminescence western blotting detection system.

### H&E staining and immunohistochemistry staining

To analyze the pathological changes in mouse skin lesions, the skin tissue was cut off and fixed with 4% paraformaldehyde in PBS buffer. The H&E is used for the evaluation of epidermal thickness. For immunochemistry, some skin paraffin sections were stained with anti-CD11b (1:100) and anti-Ly6G (1:100). The sections were imaged using a microscope slide scanner (Pannoramic 250 FLASH).

### Flow cytometry assay

BALB/c mice (female, 8 weeks old) were administered 50 µL Hlb (0.4 mg/mL) by subcutaneous injection. On the eighth day, the mice were euthanized and the skin around the hydrogel site was cut off. The immune cell infiltration in the epidermis and dermis was separated according to the standard protocol (Protocol for Flow Cytometric Detection of Immune Cell Infiltration in the Epidermis and Dermis of a Psoriasis Mouse Model). To detect the cell type, the separated cells were stained with anti-CD45, anti-CD11b, anti-CD11c, anti-F4/80, anti-NKp46, and anti-Ly6G (BioLegend) for 30 min on ice and were analyzed by flow cytometry (BD AriaII).

HaCaT cells (1 × 10^6^ cells/mL, 200 µL) were pre-treated with GW280264X (3 µM) for 30 min and then were treated with 1 µg/mL Hlb for 10 min. PS exposure was detected with a commercial kit according to the manufacturer’s instructions. Briefly, cells were harvested and washed with 1 × Binding Buffer (556547, BD Biosciences, USA), followed by incubation with Annexin V-FITC for 10 min at room temperature. Cells were then washed and resuspended with 1× Binding Buffer. Flow cytometric data were acquired on a CytoFLEX flow cytometer (Beckman Coutler, USA) and analyzed using FlowJo software.

### Transfection of HaCaT cells with ADAM17-siRNA

Small interference RNAs against ADAM17 were ordered from KeyGen BioTech, Nanjing, China. HaCaT cells of the ADAM17-siRNA groups were transfected with Lipofectamine 2,000 following the manufacturer’s instructions. The cells were collected 48 h later for western blotting analysis.

### Red-blood cell lysis assay

Anti-Hlb mAb was serial diluted and then mixed with purified recombinant Hlb protein (0.008 µg/mL). The antibody-antigen complexes were incubated with 2% (vol/vol) suspension of PBS-washed fresh sheep erythrocytes at 37°C for 1 h, followed by 4°C for 1 h. The released hemoglobin in the supernatant was collected by centrifugation and determined at 405 nm.

### Annexin V-FITC staining

HaCaT cells grown on coverslips were incubated with 1 µg/mL of Hlb proteins for 10 min. The coverslips were washed once with cold PBS and subsequently incubated with a 1:20 solution of Annexin V-FITC in Annexin-Binding-Buffer for 5 min. Cells were washed twice with Annexin-Binding-Buffer and fixed with 3% paraformaldehyde for 15 min. Coverslips were washed four times with cold PBS and mounted on microscope slides using DAPI-Fluoromount-G (Southern Biotech). Image analysis was done using a Nikon TiE-A1 confocal scanning microscope.

## SUPPLEMENTAL MATERIAL

Fig. S1 spectrum.02227-23-s0001.pdf The dose of Hlb protein used in this study shows no detectable cytotoxic effect to HaCaT or HFF-1 cells.

Fig. S2 spectrum.02227-23-s0002.pdf Hlb-induced mouse skin inflammation relies on Hlb's sphingomyelinase activity.

Fig. S3 spectrum.02227-23-s0003.pdf Detection of soluble TNFR1 in the cell culture.

Fig. S4 spectrum.02227-23-s0004.pdf Analysis of PS exposure on the outer leaflet of the cell membrane in HaCaT cells treated with or without Hlb and the ADAM17 inhibitor (GW280264X).

Fig. S5 spectrum.02227-23-s0005.pdf Detection of the inhibitory effect of αHlb mAb on the skin infection caused by *S. aureus* MW2.

Supplementantal legends spectrum.02227-23-s0006.docx Legends for Fig. S1 to S5.

ASM does not own the copyrights to Supplemental Material that may be linked to, or accessed through, an article. The authors have granted ASM a non-exclusive, world-wide license to publish the Supplemental Material files. Please contact the corresponding author directly for reuse.
