# Supplementary figures and images for: Staphylococcus aureus β-hemolysin causes skin inflammation by acting as an agonist of epidermal growth factor receptor
Source: Microbiol Spectr. 2023 Dec 7;12(1):e02227-23. doi: 10.1128/spectrum.02227-23 (PMC10783061; doi:10.1128/spectrum.02227-23)

Supplementary Fig. 1

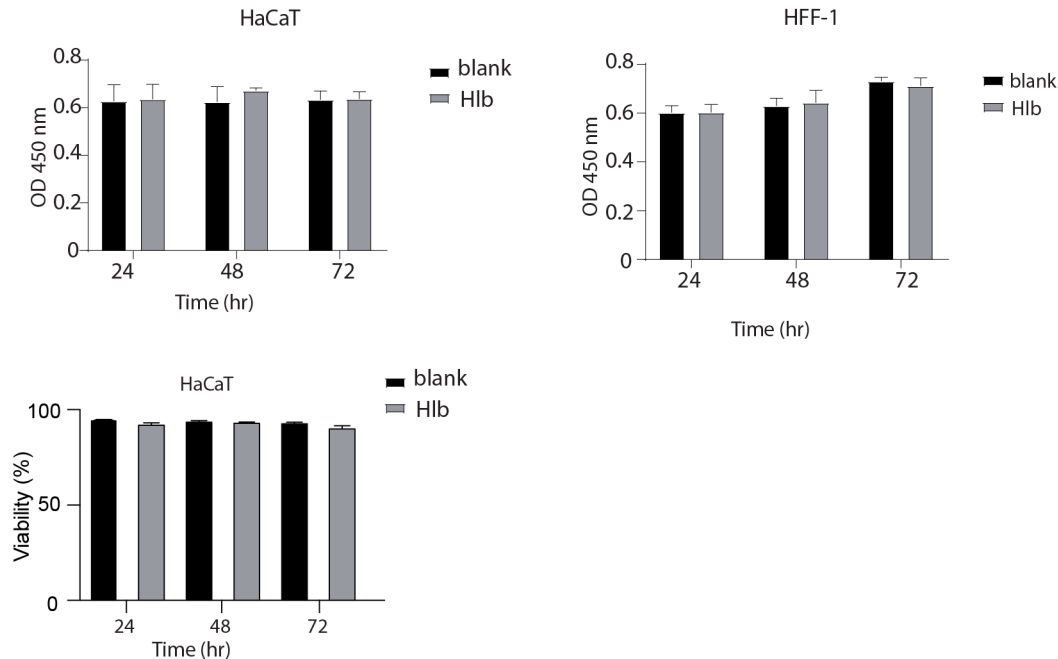

Supplement: Fig. S1 — The dose of Hlb protein used in this study shows no detectable cytotoxic effect to HaCaT or HFF-1 cells. [file spectrum.02227-23-s0001.pdf]

Supplementary Fig. 2

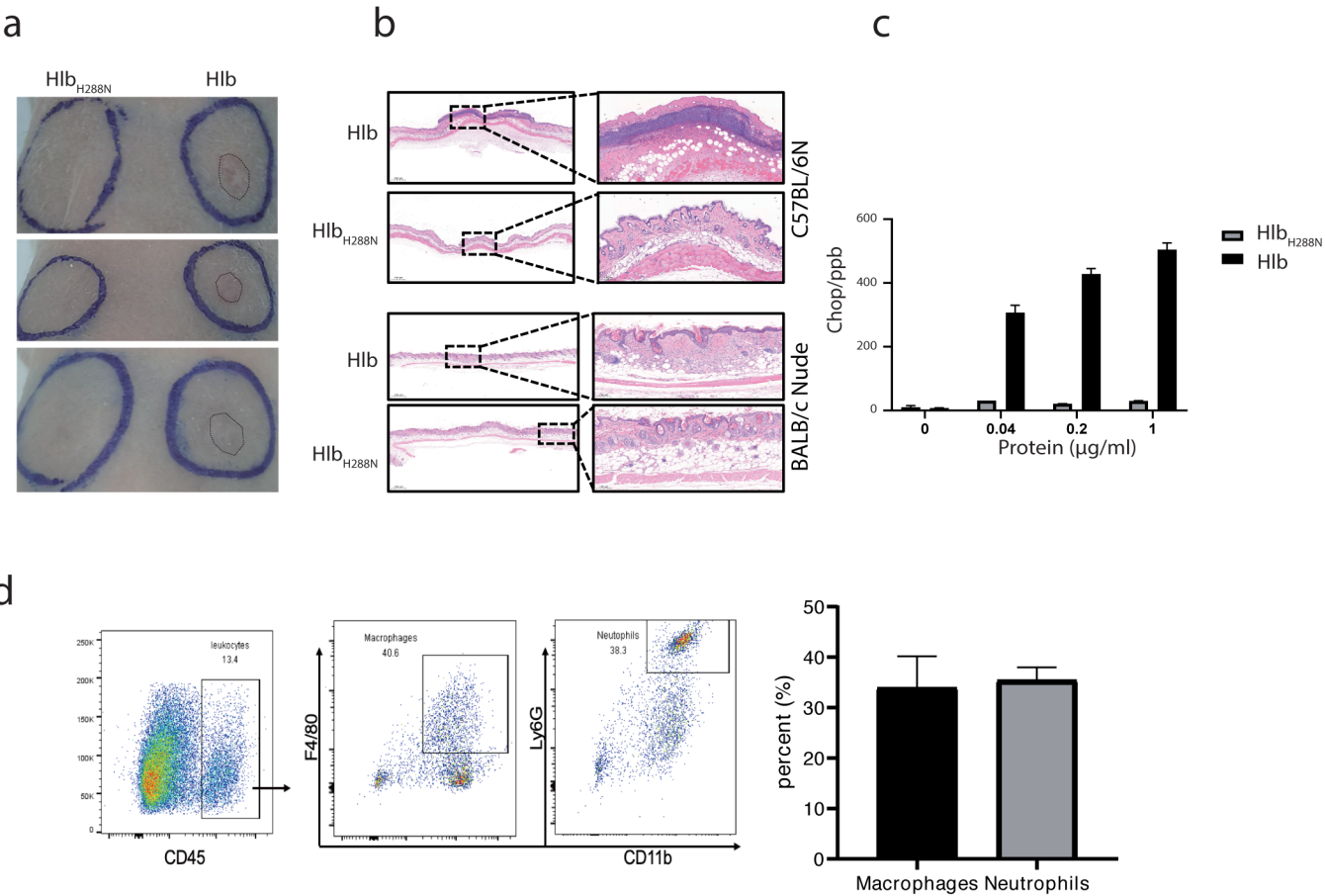

Supplement: Fig. S2 — Hlb-induced mouse skin inflammation relies on Hlb's sphingomyelinase activity. [file spectrum.02227-23-s0002.pdf]

Supplementary Fig. 3

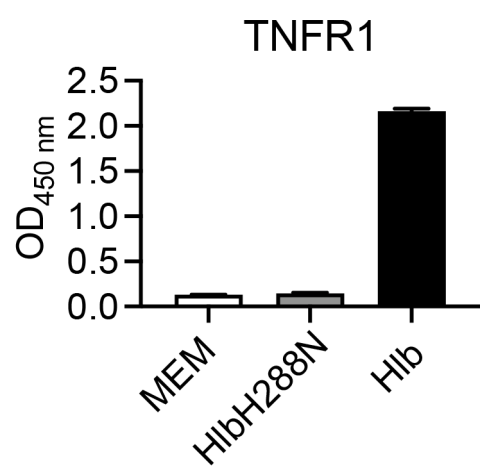

Supplement: Fig. S3 — Detection of soluble TNFR1 in the cell culture. [file spectrum.02227-23-s0003.pdf]

Supplementary Fig. 4

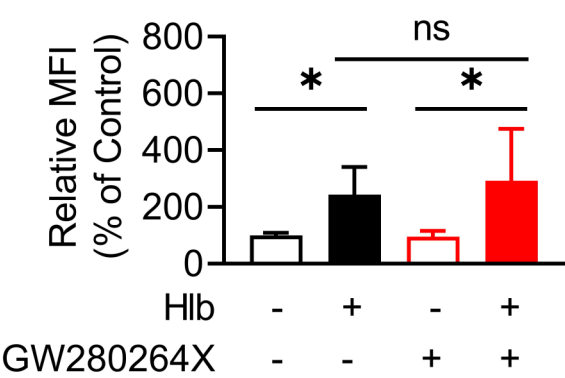

Supplement: Fig. S4 — Analysis of PS exposure on the outer leaflet of the cell membrane in HaCaT cells treated with or without Hlb and the ADAM17 inhibitor (GW280264X). [file spectrum.02227-23-s0004.pdf]

Supplementary Fig. 5

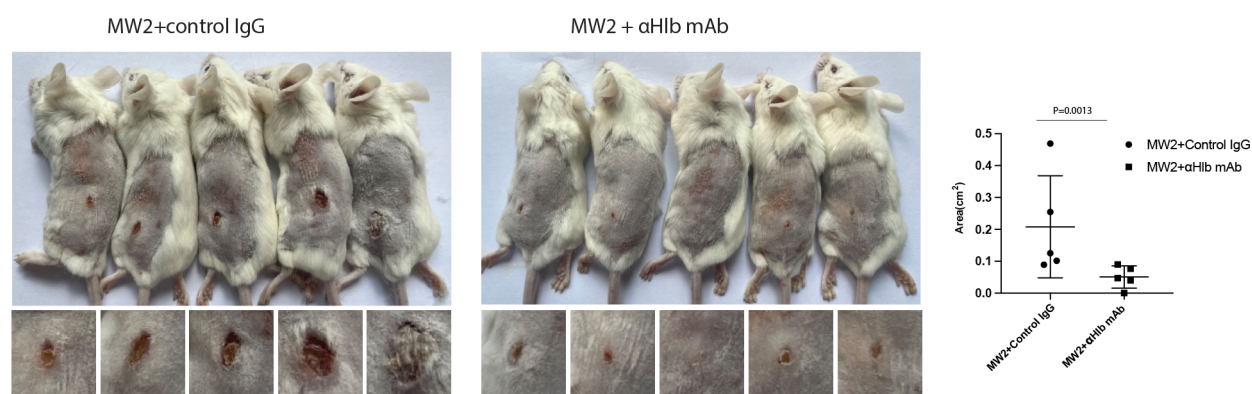

Supplement: Fig. S5 — Detection of the inhibitory effect of αHlb mAb on the skin infection caused by S. aureus MW2. [file spectrum.02227-23-s0005.pdf]
