# Supplementary material for: Staphylococcus aureus β-hemolysin causes skin inflammation by acting as an agonist of epidermal growth factor receptor
Source: Microbiol Spectr. 2023 Dec 7;12(1):e02227-23. doi: 10.1128/spectrum.02227-23 (PMC10783061; doi:10.1128/spectrum.02227-23)
Supplement: Supplementantal legends — Legends for Fig. S1 to S5. [file spectrum.02227-23-s0006.docx]

**Supplementary Fig legend**

**ADDITIONAL INFORMATION**

**Supplemental information**

Supplementary Fig.1 **The dose of Hlb protein used in this study shows no detectable cytotoxic effect to HaCaT or HFF-1 cells**

The cell viability of HaCaT or HFF-1 after incubation with Hlb protein (1 μg/ml) for 24 h, 48 h and 72 h was measured by CCK-8 assay, a sensitive colorimetric method for assessing cell viability in cell proliferation and cytotoxicity assays. HaCaT cells after Hlb-treatment were stained with AO/PI and counted using an automated imaged based cytometry method (BobBoge Automatic Cell counter).

Supplementary Fig.2 **Hlb-induced mouse skin inflammation relies on Hlb’s sphingomyelinase activity**

a)BALB/c nude mice were subcutaneously injected with Hlb_H288N_ (20 μg) or Hlb(20 μg). Sphingomyelinase-deficient Hlb mutant totally lost the ability to induce mouse skin inflammation. The skin lesion region was labeled by dashed lines. b) Pathological changes in C57BL/6N (b upper), BALB/c Nude (b lower) mice were identified by H&E staining. c)A549 cells were incubated with Hlb (1 μg/ml) or HlbH-288-N (1 μg/ml). The phosphocholine in supernatant was determined by ELISA. d) Inflammatory cells infiltrate in Hlb-injected mouse skin tissues were identified by flow cytometry.

Supplementary Fig.3 **Detection of soluble TNFR1 in the cell culture**

The release of ADAM17 substrate TNFR1 in the supernatant was measured by ELISA in HaCaT cells after incubation with HlbH288N (1 μg/ml) or Hlb protein (1 μg/ml). The data represent the mean ± standard deviation from 3 independent experiments.

Supplementary Fig.4 **Analysis of PS exposure on** **the outer leaflet of the cell membrane in HaCaT cells treated with or without Hlb and the ADAM17 inhibitor (GW280264X).**

PS exposure was determined by quantifying the relative mean fluorescence intensity (MFI), normalized to untreated HaCaT cells. The data represent the mean ± standard deviation from four independent experiments. Statistical significance was determined using Student's t-test (*p < 0.05).

Supplementary Fig.5 **Detection of the inhibitory effect of αHlb mAb on the skin infection caused by S. aureus MW2**

Mice were subcutaneous injected with S. aureus MW2 in the presence of αHlb mAb or unrelated mouse control IgG. Mice infected with MW2 in the presence of αHlb mAb developed significantly smaller lesions compared with control IgG antibody. The lesion sizes were measured and shown as mean ± standard deviation.
